# Systematic analysis of evaluation metrics for deep learning-based surgical phase recognition in laparoscopic cholecystectomy

**DOI:** 10.3389/frai.2026.1873219

**Published:** 2026-07-08

**Authors:** Gizem Özkurt, Flakë Bajraktari, Peter P. Pott

**Affiliations:** Institute of Medical Device Technology, University of Stuttgart, Stuttgart, Germany

**Keywords:** artificial intelligence, evaluation, metrics, offline models, online models, surgical phase recognition, systematic analysis

## Abstract

Evaluation metrics are essential for assessing the performance of AI models and enabling comparability across different approaches. However, in surgical phase recognition, their application remains inconsistent. This study provides a systematic analysis of evaluation metrics used in this field, aiming to support standardization and encourage more explicit explanation of metric selection. A systematic literature review was conducted following the PRISMA framework, covering publications from 2016 to 2025. In total, 50 studies were identified and analyzed with respect to the evaluation metrics applied, with particular focus on relaxed boundaries and the distinction between online and offline models. The results show that accuracy, precision, and recall are the most frequently used metrics, followed by the jaccard index. Since around 2023, an increasing use of segment-based metrics can be observed, reflecting a growing emphasis on temporal dynamics. No significant differences in metric selection between online and offline approaches were identified. Furthermore, many studies do not provide explicit explanation for their choice of evaluation metrics. Instead, they often rely on commonly used metrics or practices adopted from previous work. Additionally, results obtained with relaxed boundaries tend to exhibit higher standard deviations compared to those without relaxed boundaries. Based on these findings, it is recommended to more explicitly align evaluation metrics with model objectives and to further investigate the distinction between online and offline settings. Adaptations of the *f*_1_-score, such as the *f*_β_-score, may provide a more flexible evaluation framework. Furthermore, the Matthews Correlation Coefficient represents a promising complementary metric, particularly for multi-class settings, if appropriately normalized. Future work should focus on clearly explaining metric selection and consistently reporting relaxed boundaries to improve transparency, comparability, and interpretability in surgical phase recognition.

## 1 Introduction

The continuous digitalization in surgery is leading to an increasing integration of modern systems and devices into clinical practice. In particular, operating rooms are evolving into increasingly complex environments that support surgical staff in performing precise and successful procedures (Demir et al., 2023). To support the modernization of operating rooms and to improve surgical capabilities—and consequently patient outcomes—Computer-Assisted Surgery (CAS) is being introduced (Zhang et al., 2021). CAS has developed significantly in recent years and provides essential support to surgeons in managing complex surgical situations. A central component of CAS is Surgical Workflow Recognition (SWR), which aims to automatically recognize and analyze events within a surgical procedure (Li et al., 2024).

In general, SWR can be performed either online or offline (Li et al., 2024). In online recognition, only information from previous time steps is used, enabling real-time analysis and practical application in the surgical environment (Funke et al., 2023; Liu et al., 2023). This approach supports, among other things, the monitoring of surgical workflows, error prevention, and the assistance of the operating team through warning systems, structured process guidance, and decision support (Demir et al., 2023; Funke et al., 2023; Liu et al., 2023). In contrast, offline recognition can utilize all available information from the complete video, including future information (Funke et al., 2023). It is particularly suitable for training and documentation purposes, such as the automated generation of surgical reports or the evaluation of surgical performance (Demir et al., 2023; Li et al., 2024).

**Figure 1:**
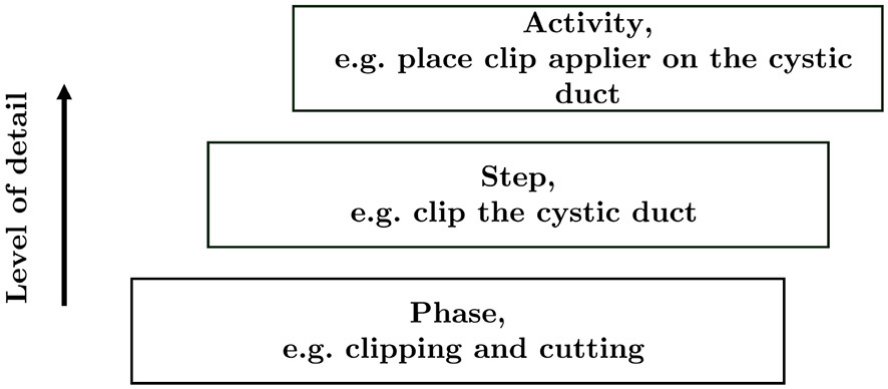
Hierarchical structure of surgical procedures, illustrated with examples. The arrow indicates increasing levels of detail.

One important research area is automated surgical phase recognition (SPR), which enables systematic monitoring of surgical processes, improved staff planning, and real-time support for clinical personnel. For the structured analysis of surgical procedures, the terms surgical phases, steps, and activities are commonly used (Demir et al., 2023). Surgical phases represent the higher-level stages of an intervention and include key segments such as clipping and cutting. Surgical steps refer to more fine-grained processes within these phases that are necessary to achieve surgical objectives, like clipping the cystic duct (Li et al., 2024). Surgical activities represent the smallest units of action during a procedure, such as placing clip applier on the cystic duct (Demir et al., 2024). This hierarchical structure of surgical workflows is illustrated in Figure 1, where the hierarchy becomes more refined and detailed as it moves up the arrow. Furthermore, individual examples are included within the visualization to support understanding.

To objectively evaluate the performance of such systems, evaluation metrics are employed. These metrics allow for quantifying the strengths of algorithms, assessing model performance, and enabling comparisons across studies (Li et al., 2024).

In this work, the focus is on automated SPR. This task is of particular importance for computer-assisted surgical systems, as it addresses key requirements of the modern operating room, including workflow monitoring, surgical scheduling, and improved coordination between surgical teams. Consequently, it provides substantial added value for CAS (Yi et al., 2023).

Current research in SPR primarily focuses on minimally invasive surgery, a technique widely adopted due to its numerous advantages. These include reduced blood loss, shorter recovery times, and a lower risk of infection (Lee et al., 2024). A representative procedure in this domain is cholecystectomy. With more than 500,000 laparoscopic or robot-assisted cholecystectomies performed annually in the United States, this procedure is among the most common surgical intervention worldwide (Ban et al., 2022). Laparoscopic cholecystectomy (LC) is a minimally invasive procedure for gallbladder removal and is widely performed in clinical practice (Cheng et al., 2022). Its advantages include reduced pain, lower blood loss, and the avoidance of large open incisions (Kadkhodamohammadi et al., 2022). During the procedure, surgical instruments are inserted into the abdominal cavity through small incisions using a laparoscope (Pradeep and Sinha, 2021). However, LC is not fully standardized. In particular, cases involving acute cholecystitis, severe inflammation, or fibrotic tissue can significantly increase procedural complexity. Inflammatory processes impair intraoperative orientation and elevate the risk of postoperative complications. Additionally, the duration of surgery varies considerably and depends strongly on the surgeon's experience and skill (Cheng et al., 2022). Furthermore, errors frequently occur due to incorrect instrument usage within inappropriate phases or an incorrect sequence of procedural steps, especially among less experienced surgeons (Putalapattu, 2022). Against this background, SPR represents an important approach to structuring and standardizing LC, as well as reducing potential sources of error.

**Figure 2:**
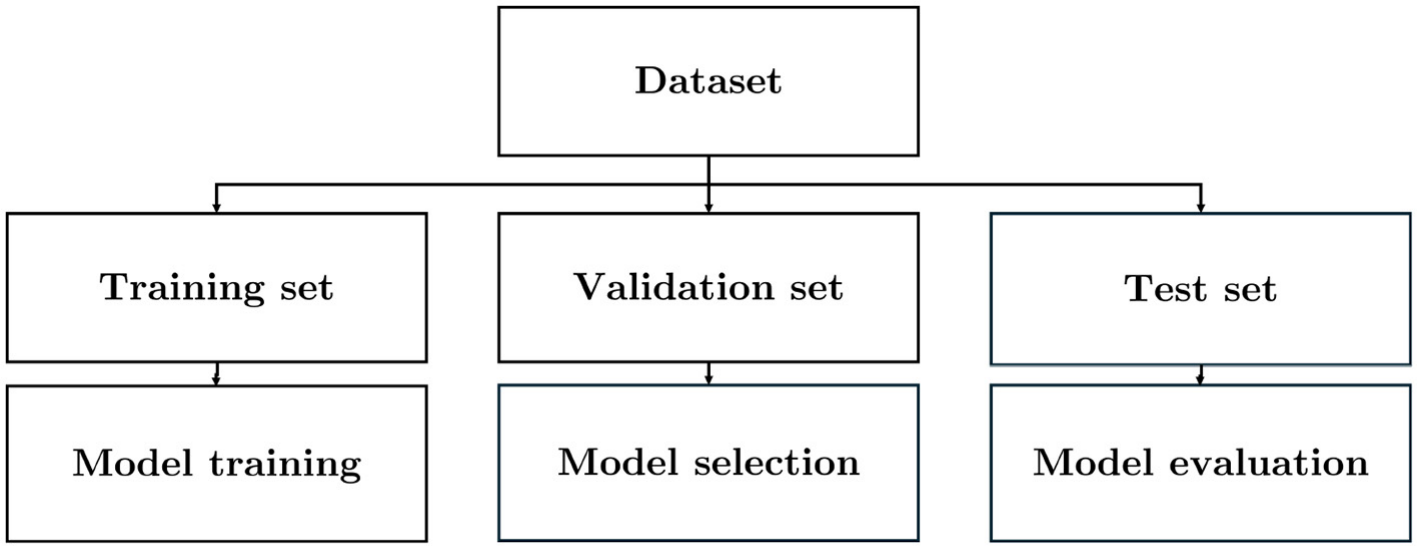
Schematic representation of the split of a dataset into training, validation, and test sets, along with their functions.

Overall, surgical video recordings serve a valuable role in the operating room. In this context, these recordings can be structured as datasets and categorized into three main groups, as described Figure 2 (De Backer et al., 2023). A training set is used to train the model, a validation set is used to monitor performance on unseen data and select a model, and a test set is used for final model evaluation (Hicks et al., 2022).

**Figure 3:**
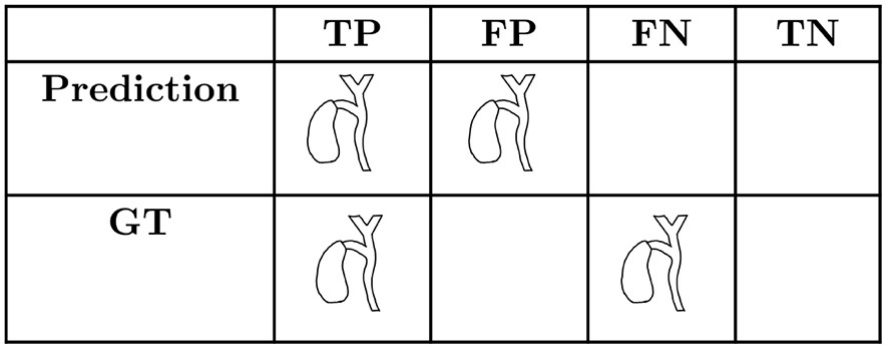
Comparison of predictions and GT with classification into TP, FP, FN, and TN. The presence of the gallbladder represents a surgical phase.

Evaluating model performance is a fundamental component of the machine learning pipeline, as it enables the quantification of algorithmic performance and facilitates comparison with existing approaches using defined evaluation metrics. Many evaluation metrics are derived from the so-called confusion matrix, which summarizes the number of correctly and incorrectly classified instances (Li et al., 2024; Naidu et al., 2023). To provide an intuitive understanding of its entries, Figure 3 illustrates the possible outcomes by indicating whether the gallbladder was detected or not, and whether the prediction matches the Ground Truth (GT). The model predicts the presence of the gallbladder in an image, which represents a surgical phase.

The confusion matrix distinguishes between the following cases:

- **True positive (TP):** the gallbladder is present and correctly detected.
- **True negative (TN):** no gallbladder is present, and the model correctly identifies its absence.
- **False positive (FP):** a gallbladder is incorrectly detected although none is present.
- **False negative (FN):** the existing gallbladder is not detected by the model (Hicks et al., 2022).

Based on the confusion matrix and its entries, evaluation metrics are computed, which are described in detail in the following.

- **Accuracy:** represents the proportion of correctly classified instances—both correctly identified positive and negative cases—out of all predictions made by the model. The metric is bounded within the range [0, 1], where a value of 1 indicates perfect classification and 0 indicates completely incorrect classification across all positive and negative instances (Hicks et al., 2022).
- **Precision:** describes the proportion of instances that are predicted as positive and are indeed true positives. This metric therefore reflects the reliability of a model. A high precision value indicates that the model produces few false positive predictions, whereas a low precision value reflects a higher number of incorrect positive predictions. Precision is bounded within the range [0, 1] (Li et al., 2021).
- **Recall:** also known as sensitivity, measures the proportion of actual positive cases that are correctly identified by the model. It therefore reflects the completeness of detection. There is often a trade-off between precision and recall, where an increase in one may lead to a decrease in the other (Funke et al., 2023). Recall is particularly important in medical applications, as it is desirable to miss as few positive cases as possible, which corresponds to achieving a high recall value. This metric is also bounded within the range [0, 1] (Hicks et al., 2022).
- ***F*_1_-Score:** represents the harmonic mean of precision and recall. It is bounded within the range [0, 1], where a value of 1 indicates perfect precision and recall, and 0 indicates the absence of both (Hicks et al., 2022). The generalized formulation of the f-measure includes a parameter β, which determines the relative weighting between precision and recall. When β is smaller than 1, precision is weighted more strongly, whereas β is larger than 1 places greater emphasis on recall (Sasaki, 2007).
- **Jaccard-Index:** Another commonly used evaluation metric is the jaccard index, also known as Intersection over Union (IoU) (Li et al., 2021). It measures the similarity between predicted and GT segments by comparing their overlap relative to their union. The metric is bounded within the range [0, 1].

For evaluation, temporal tolerance windows can be applied to account for minor deviations at phase transitions. These so-called relaxed boundaries (RB) vary across studies and are typically defined in seconds (Funke et al., 2023).

However, the literature on SPR reveals a high degree of inconsistency in the selection and application of evaluation metrics. Different studies employ varying combinations of metrics without explaining their choices. This hinders the comparability of results and reduces the interpretability of reported model performance. Moreover, there is a risk that future evaluation practices may develop without a clear objective or standardized basis.

In this work, evaluation metrics are not formally derived or mathematically defined, as these definitions are already available in existing literature (Funke et al., 2023). While previous studies report the use of evaluation metrics, they do not systematically analyze their application. In particular, prior work focuses on definitions, calculations, and inconsistencies of metrics, whereas aspects such as usage patterns, explanation strategies, temporal trends, and the relationship between model settings (online/offline) and metric selection remain largely unexplored. Therefore, the aim of this work is to systematically analyze the use of evaluation metrics in SPR. To this end, the metrics reported in the literature are cataloged and analyzed with respect to their frequency, temporal development, and typical combinations. Furthermore, the relationship between specific evaluation metrics and scenarios is investigated. The results of this analysis aim to improve transparency and comparability in future studies and to provide a structured basis for the selection of appropriate evaluation metrics in SPR.

## 2 Methods

A methodological approach based on a systematic literature review following the PRISMA framework was adopted in this study. The objective was to identify studies published between 2016 and 2025 that address evaluation metrics in SPR. From the selected studies, structured Tables were created to capture key information on the evaluation metrics used, as well as the application of RB. This approach enables the investigation of how evaluation metrics and their combinations have evolved over time and which trends can be identified. In addition, the relationship between the explanation of metric selection and the metrics used is examined.

### 2.1 Literature search strategy

**Figure 4:**
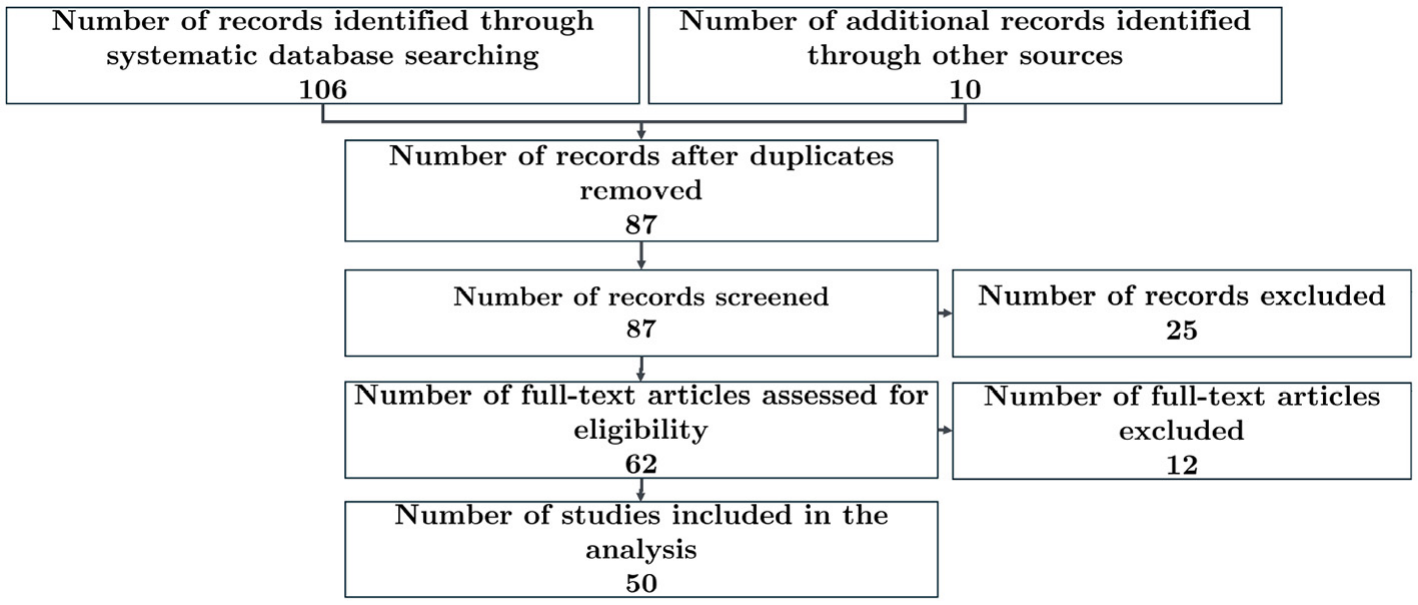
PRISMA flow diagram of the systematic literature selection process.

A structured literature selection is essential to accurately represent the current state of research in SPR. To identify relevant scientific publications for the analysis of existing evaluation metrics, a systematic literature review was conducted based on the Preferred Reporting Items for Systematic Reviews and Meta-Analyses (PRISMA) framework (Mishra and Mishra, 2023). The workflow of the PRISMA methodology is illustrated in Figure 4.

The literature search was conducted using the databases IEEE Xplore, Google Scholar, and PubMed, as these provide comprehensive coverage of research in SPR. There was no database-specific use of search strings, and the literature search was carried out from January to March 2025. Two reviewers independently screened the titles and abstracts and evaluated them according to the established inclusion and exclusion criteria. Studies were subsequently either advanced to the next stage of the review process or excluded from further consideration. During the full-text screening stage, the first author reviewed the contents of the eligible articles and performed the subsequent analysis under the supervision of the second author. Any disagreements regarding eligibility assessment or data extraction were addressed through weekly discussions among the whole research team until consensus was reached.

To ensure a focused and comprehensive search, Boolean operators were applied to refine the search queries. This approach ensured that relevant articles from related research domains were systematically identified and included in the analysis. Search terms were selected based on their relevance to the research topic. Similar terms such as Surgery, Surgical Procedure, and Surgical were grouped and combined using the OR operator within parentheses. The Boolean search queries used in this study were as follows:

- (Evaluation Metrics OR Performance Metrics) AND (Video OR Surgery OR Surgical Procedure OR Phase Recognition OR Surgical Workflow Recognition) OR (Surgical Phase Recognition) AND (Cholec80 OR Laparoscopic Cholecystectomy).
- (Evaluation Metrics Time) AND (Surgical Phase Recognition Evaluation Metric OR Evaluation Metrics Surgery OR Surgical Workflow Recognition OR Surgical Workflow Analysis OR Surgical Workflow Detection OR Surgical Step Recognition).

For the selection of relevant scientific articles, the following criteria were applied: The studies had to be published in either English or German. Accordingly, only full-text articles were considered and included in the analysis. The publication period was restricted to studies published between 2016 and 2025, as this timeframe defines the scope of this work. Furthermore, only studies based on supervised learning approaches were included. Only articles that were primarily based on the Cholec80 dataset were included in the analysis. Cholec80 represents one of the most widely used benchmark datasets in surgical phase recognition and forms the basis of the majority of articles included in this review. Furthermore, different datasets may involve varying benchmark settings, phase definitions, annotation protocols, and phase durations, which could introduce additional variability and complicate the interpretation of trends in evaluation practices. Therefore, the analysis was intentionally restricted to Cholec80-based articles. No distinction was made regarding whether additional external validation datasets were used. Studies with clearly defined training, validation, and test splits were included in the analysis. Studies were required to describe the evaluation metrics and model settings. Consequently, articles employing unsupervised learning methods, as well as publications prior to 2016, were excluded. Twelve full-text articles were excluded. The exclusion was due to several reasons: some articles were conducted on other, less commonly used datasets instead of Cholec80 and were therefore excluded to ensure clarity and consistency. One preprint was included only for background purposes, as it constitutes a fundamental reference in the field of SPR. To the best of current knowledge, it is the only study that comprehensively addresses evaluation metrics from theoretical, technical, and mathematical perspectives and provides a detailed discussion of relaxed boundaries. All other included articles are peer-reviewed publications that satisfied the predefined inclusion criteria and were subjected to the screening process. In total, 50 articles were included in the analysis. However, the distribution of studies over the years shows noticeable variation, and a uniform annual distribution could not be achieved. For example, no relevant studies were identified for the years 2018 and 2025; therefore, those years was excluded from graphical and tabular representations. Studies that considered both modes (online and offline) or included multiple models—some using RB and others not—were analyzed and reported separately.

It was important whether studies reported clearly defined training, validation, test splits and specified online or offline settings. Furthermore, studies should describe the evaluation metrics used, including the application of relaxed boundaries. These criteria were considered essential to ensure comparability of the reported results. Public code availability and the use of external validation datasets were not used as inclusion criteria and were therefore not assessed.

An overview of all studies included in this systematic review is provided in Table 1, which lists the publication year, authors, datasplit, the use of RB, model type (online/offline), and the evaluation metrics used. All analyses conducted in this work are based on the models presented in this table. No use of RB is indicated by “–”, whereas available use of RB is marked with “+.”

**Table 1:** Comparison of SPR models, grouped by online and offline settings.

Year	Model name	References	Datasplit	RB	A	P	R	*F*_1_	J	Seg. Edit	@10	@25	@50	*F*_1*AVG*_
Online														
2016	EndoNet	Twinanda et al. (2017)	40/40	–	81.70 ± 4.20	73.70 ± 16.10	79.60 ± 7.90	–	–	–	–	–	–	–
2017	SV–RCNet	Jin (2018)	40/40	+	85.30 ± 7.30	80.70 ± 7.00	83.50 ± 7.50	–	–	–	–	–	–	–
2019	OHFM	Yi and Jiang (2019)	40/40	+	87.30 ± 5.70	–	–	–	67.00 ± 13.30	–	–	–	–	–
2020	TeCNO	Czempiel et al. (2020)	40/8/32	–	88.56 ± 0.27	81.64 ± 0.41	85.24 ± 1.06	–	–	–	–	–	–	–
2020	MTRCNet–CL	Jin et al. (2020)	40/40	+	89.20 ± 7.60	86.90 ± 4.30	88.00 ± 6.90	87.40	–	–	–	–	–	–
2021	OperA	Czempiel et al. (2021)	48/12/20	–	91.26 ± 0.64	–	–	84.49 ± 0.64	–	–	–	–	–	–
2021	Trans–SVNet	Gao et al. (2021)	40/40	+	90.30 ± 7.10	90.70 ± 5.00	88.80 ± 7.40	–	79.30 ± 6.60	–	–	–	–	–
2021	TMRNet	Jin et al. (2021)	32/8/40	+	90.10 ± 7.60	90.30 ± 3.30	89.50 ± 5.00	–	79.10 ± 5.70	–	–	–	–	–
2021	NETE (GRU)	Yi et al. (2023)	40/40	–	90.8 ± 7.0	–	85.6 ± 10.0	–	75.5 ± 11.10	–	–	–	–	–
2021	NETE TCN	Yi et al. (2023)	40/40	–	92.80 ± 5.0	–	87.5 ± 8.30	–	78.7 ± 9.40	–	–	–	–	–
2022	–	Chen et al. (2022)	40/40	–	91.4	85.4	86.3	–	75.4	–	–	–	–	–
2022	SAHC	Ding and Li (2022)	32/8/40	+	91.80 ± 8.10	90.30 ± 6.40	90.00 ± 6.40	–	81.20	–	–	–	–	–
2022	PATG	Kadkhodamohammadi et al., 2022	40/40	–	93.77 ± 0.44	89.79 ± 0.79	89.11 ± 0.65	88.22 ± 0.18	–	–	–	–	–	–
2022	ARST	Zou et al. (2023)	40/8/32	–	88.46 ± 6.81	84.93 ± 7.83	85.05 ± 7.24	73.16 ± 10.17	–	–	–	–	–	–
2023	TUNeS	Funke et al. (2025)	32/8/40	–	92.22 ± 0.7	–	–	–	78.30 ± 1.10	–	–	–	–	–
2023	TUNeS	Funke et al. (2025)	32/8/40	+	93.90 ± 5.0	–	–	–	84.20 ± 8.30	–	–	–	–	–
2023	SKiT	Liu et al. (2023)	40/40	+	93.40 ± 5.20	90.90	91.80	–	82.60	–	–	–	–	–
2023	SKiT	Liu et al. (2023)	40/40	–	92.50 ± 5.10	84.60	88.50	–	76.70	–	–	–	–	–
2023	LoViT	Liu et al. (2025)	40/40	–	91.50 ± 6.10	83.10	86.50	–	74.20	–	–	–	–	–
2023	TSTNet–PRA	Pan et al. (2023)	40/40	–	92.80 ± 8.60	90.50 ± 8.50	90.70 ± 8.70	–	–	–	–	–	–	–
2023	LAST	Tao et al. (2023)	40/40	–	93.12 ± 4.71	89.25 ± 5.49	90.10 ± 5.45	81.11 ± 7.62	–	–	–	–	–	–
2023	CMTNet	Yue et al. (2023)	40/40	–	92.4 ± 5.60	89.8 ± 6.80	91.0 ± 4.80	–	80.0 ± 9.70	–	–	–	–	–
2023	CMTNet	Yue et al. (2023)	40/40	+	92.9 ± 5.9	90.1 ± 7.10	92.0 ± 4.40	–	81.50 ± 10.4	–	–	–	–	–
2023	C–ECT	Zhang et al. (2023)	40/40	–	94.67 ± 4.32	92.66 ± 5.38	91.11 ± 7.28	–	84.02 ± 8.86	53.00	65.93	65.68	60.52	64.04
2024	–	Zhang S. et al. (2024)	40/40	–	93.6 ± 5.10	90.80 ± 5.40	91.80 ± 3.60	–	83.50 ± 6.20	–	–	–	–	–
2024	MS–ASCT	Zhang et al. (2024a)	40/40	–	93.58 ± 0.13	88.90 ± 0.41	88.20 ± 0.34	–	–	–	–	–	–	–
Offline														
2016	EndoNet	Twinanda et al. (2017)	40/40	–	92.00 ± 1.40	84.80 ± 9.10	88.30 ± 5.50	–	–	–	–	–	–	–
2021	–	Ban et al. (2021)	40/40	–	90.80	85.30	82.70	84.00	–	–	–	–	–	–
2023	TuNeS	Funke et al. (2025)	32/8/40	–	94.20 ± 0.60	–	–	–	82.30 ± 1.50	–	–	–	–	–
2023	TuNeS	Funke et al. (2025)	32/8/40	+	96.40 ± 2.20	93.60 ± 7.00	93.90 ± 5.40	–	–	–	–	–	–	–
2023	SF–TMN (MS–TCN)	Zhang et al. 2024b)	40/40	–	93.58 ± 4.70	92.18 ± 4.54	90.66 ± 6.52	–	83.03 ± 6.33	86.56	–	–	–	88.07
2023	SF–TMN (Transformer)	Zhang et al. (2024b)	40/40	–	95.43 ± 3.98	92.40 ± 5.31	93.41 ± 4.41	–	86.14 ± 6.61	87.21	–	–	–	88.23
2024	MS–AST	Zhang et al. (2024a)	40/40	–	95.17 ± 0.07	90.41 ± 0.20	91.85 ± 0.13	–	–	–	–	–	–	–
2024	–	Zhang S. et al. (2024)	40/40	–	93.60 ± 5.10	90.80 ± 5.40	91.80 ± 3.60	–	83.50 ± 6.20	–	–	–	–	–

Datasplit denotes Training/Validation/Test or Training/Test. A denotes accuracy, P precision, R recall, *F*_1_ the *f*_1_-score, J the jaccard index, Seg. Edit the segmental edit score, @10/@25/@50 indicate segmental *f*_1_-score at different thresholds, and RB refers to relaxed boundary.

This paper aims to address the research question of how evaluation metrics and their combinations have evolved over time and which trends can be identified. Furthermore, it examines which evaluation metrics are suitable for different model settings and application scenarios in SPR, with the goal of contributing to a more standardized assessment of AI models.

### 2.2 Additional search for cross-domain metrics

In addition to the primary literature search, an exploratory search was conducted without applying the predefined inclusion and exclusion criteria. The objective was to identify evaluation metrics from other domains that could potentially be applied to SPR.

Through this search, the Matthews Correlation Coefficient (MCC) was identified. MCC has gained widespread adoption in machine learning because of its properties in the presence of imbalanced class distributions. The metric is defined within the range [–1, 1], where a value of 1 indicates perfect prediction, 0 corresponds to random prediction, and –1 represents complete disagreement between prediction and GT (Hicks et al., 2022).

## 3 Results

In this section, the evaluation metrics reported in the literature, their frequencies, combinations, and the use of RB are presented. First, the explanation provided in the studies for the selection of evaluation metrics are analyzed.

**Figure 5:**
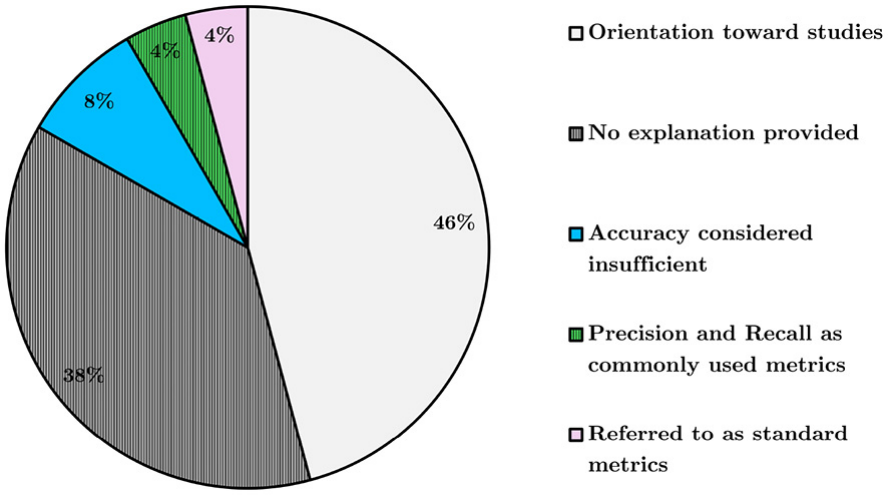
Distribution of reported explanations for the selection of evaluation metrics.

Figure 5 shows the distribution of explanations provided for the selection of evaluation metrics. 46 % of studies refer to previously published work when choosing their evaluation metrics. In several cases of 38 %, no explicit explanation for the selection of metrics is provided. Some studies state that accuracy alone is not sufficient, while others highlight that precision and recall are commonly used and established evaluation metrics.

Overall, the majority of the analyzed studies do not provide publicly available code. The publicly available implementations of the models consistently apply a temporal tolerance window of 10 s. However, neither the corresponding code nor the associated publications provide a clear explanation for the choice of this specific window or its scientific rationale. None of the analyzed studies explicitly address this aspect (Jin, 2018; Jin et al., 2021; Ding and Li, 2022; Yue et al., 2023).

**Figure 6:**
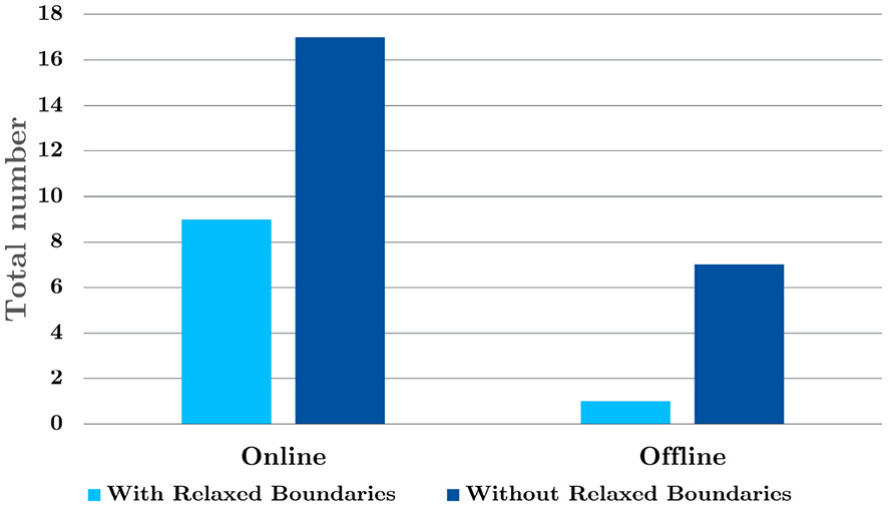
Distribution of studies with and without RB in online and offline models.

As described in the introduction, the analyzed models are categorized into online and offline SPR. Figure 6 shows the distribution of studies with respect to the use of RB. The majority of studies do not apply RB. Specifically, 17 studies do not use RB, whereas nine studies apply them. In contrast, for offline models, only one study applies RB, while seven studies do not use RB.

Based on Table 1, the following analysis examines whether the application of RB exhibits a temporal pattern within the analyzed studies from 2016 to 2025. It is important to note that the number of studies varies across years. First, the set of studies operating in the online mode is considered. For the period up to and including 2021, a total of ten studies are identified, of which five apply RB, while the remaining five do not. For the period from 2022 to 2025, the analysis includes 16 studies in the online setting. Of these, only four studies apply RB, whereas the majority (12 out of 16) do not. This suggests a decreasing use of RB in more recent studies. A similar pattern can be observed in the offline setting. Across the entire period from 2016 to 2024, studies without RB clearly predominate. Only a single study in 2023 employs RB. In general, Table 1 shows that models using RB generally exhibit higher standard deviations. As an example, two studies can be considered that evaluate both online and offline settings with and without RB. When comparing the results in terms of accuracy and jaccard index scores, a higher standard deviation can be observed for the results reported in the articles that employ RB (Funke et al., 2025; Yue et al., 2023).

**Figure 7:**
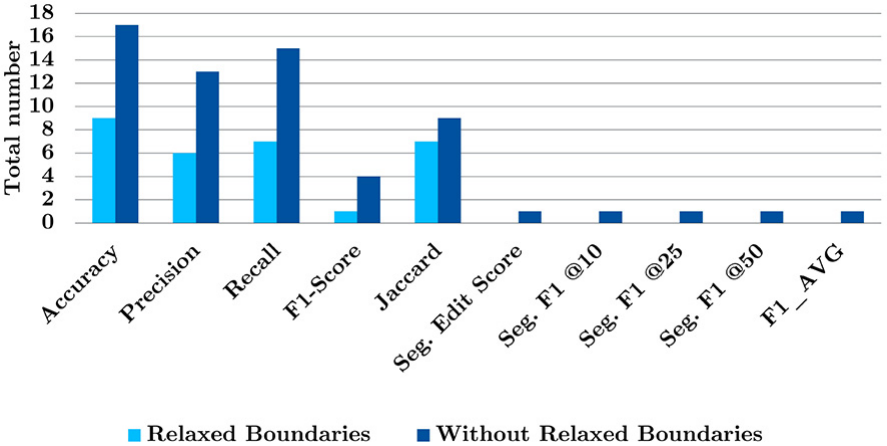
Distribution of the evaluation metrics used for online models.

Figure 7 shows the distribution of evaluation metrics for online models. The x-axis represents the individual evaluation metrics, and the y-axis indicates the total number of occurrences.

Various evaluation metrics are used in the literature to assess SPR, with results commonly reported in tabular form. Typically, the results of the respective study are highlighted in comparison to baseline or competing methods. In addition, it is often indicated whether RB are applied in both the proposed method and the compared approaches. The reported evaluation metrics are frequently accompanied by standard deviations. Overall, different metrics are presented across studies to enable comparison of model performance. In this figure, RB are applied primarily in combination with accuracy, precision, recall, *f*_1_-score, and the jaccard index in online models. No use of RB is observed for segment-based evaluation metrics. With respect to frequency, RB are most commonly used in combination with accuracy, precision, recall, and the jaccard index, followed by the *f*_1_-score. Overall, approaches without RB are reported more frequently.

**Figure 8:**
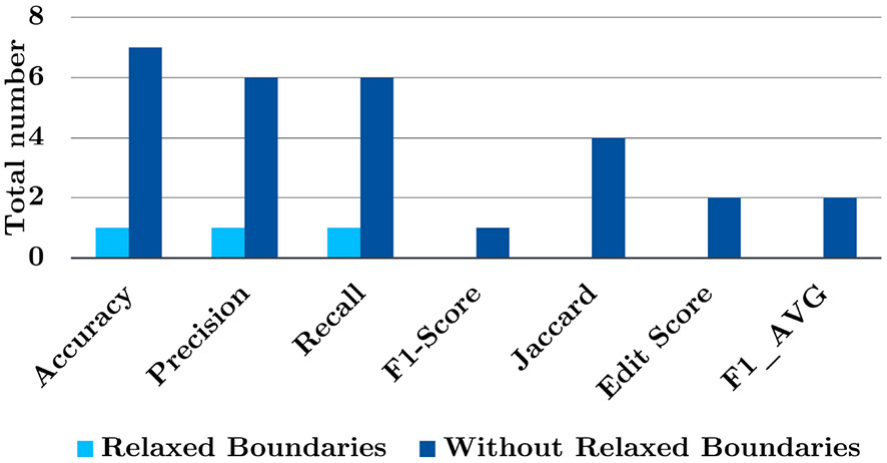
Distribution of the evaluation metrics used for offline models.

In Figure 8, fewer evaluation metrics are reported for offline models. The use of RB is also less frequent. RB are applied in combination with accuracy, precision, recall. The most frequently reported evaluation metrics without RB are accuracy, precision, recall, and jaccard index. No use of segment-based *f*_1_-score (@10, @25, @50) is reported in the analyzed studies.

As shown in Table 1, the combination of accuracy, precision, and recall is the most frequently reported set of metrics in online studies across all years.This combination is extended in some studies by additional metrics such as the jaccard index or *f*_1_-score. From 2023 onwards, an increase in the number of combined evaluation metrics is observed, including the integration of segment-based metrics. *F*_1_-score and the jaccard index are rarely reported together within the same metric combination. For offline studies, the combination of accuracy, precision, and recall is also most frequently reported. This observation is supported by the fact that in four out of five identified metric combinations, all three metrics are used together, while in the remaining combination, at least accuracy is included. In contrast, metrics such as *f*_1_-score, the jaccard index or segment-based metrics are only reported in a limited number of studies. Furthermore, accuracy, precision, and recall are the only metrics reported in studies that apply RB, whereas other metric combinations are not associated with the use of RB.

Furthermore, Table 1 allows an examination of whether the employed data splits are associated with the evaluation metrics. No clear relationship can be observed. Although the 40/40 split is the most frequently used configuration, but studies are employing this split report different combinations of evaluation metrics. Therefore, the choice of metrics does not appear to be determined by the selected data split.

The studies also address the properties as well as limitations, which are described in more detail below.

- **Accuracy:** is easy to compute and interpret, it can be misleading in the presence of class imbalance and may obscure different types of errors.
- **Precision:** ignores missed positive cases, which is why it should be combined with recall.
- **Recall:** minimizes missed positives but may increase false positives and should therefore be balanced with precision.
- ***F*_1_-score:** can be useful for imbalanced datasets, as it balances false positives and false negatives and is therefore more informative than accuracy alone. However, this metric assigns equal weight to different types of errors and is therefore unsuitable when error costs differ (Terven et al., 2025).

Across the studies analyzed in this SPR that are based on the Cholec80 dataset, no application of the MCC evaluation metric can be found.

## 4 Discussion

### 4.1 Evaluation metrics

The following section interprets the use of evaluation metrics and subsequently addresses the corresponding research question. As illustrated in Figure 5, many studies rely on evaluation metrics that have been used in previous work. This practice may contribute to an implicit standardization of evaluation metrics over time within the field of SPR. As a result, many studies do not provide explicit explanations for their choice of metrics. This may indicate that evaluation metrics are often adopted without critical reflection, potentially reinforcing their role as de facto standards. Consequently, the relationship between the explanation and the selection of evaluation metrics may lie in the fact that studies inherit metric choices from prior work without explicitly explaining their suitability. This lack of explanation makes direct comparisons between studies challenging. Without a clear understanding of the rationale behind metric selection, there is a risk of comparing results that are not methodologically equivalent. As a result, reported metric values may differ in ways that are not directly comparable, potentially leading to misleading conclusions.

As introduced in Section 1, evaluation metrics serve different purposes and capture different aspects of model performance. Therefore, future research should more carefully consider which evaluation objectives are most relevant for SPR models. Depending on the application scenario, and intended use, evaluation metrics should be selected deliberately, and their choice should be explained. This would enable more meaningful comparisons between studies and improve the interpretability of reported results.

The issue of standardization is also reflected in the use of evaluation metrics across online and offline SPR. As shown in Figure 6, the majority of the analyzed studies focus on online models. This may be explained by the ability of these models to provide real-time analysis, thereby supporting the surgical team in error prevention and decision-making processes. In this context, online models also tend to make more frequent use of RB. This may be attributed to the need to tolerate uncertainties in real-time settings, such as varying camera angles, occlusions due to blood, or rapid movements in the operating room. These factors can lead to imprecise detection of phase transitions, making strict evaluation less suitable. The use of RB may therefore allow for a more tolerant assessment of model performance under such conditions. However, it should be noted that the number of studies differs between online and offline models, which limits the ability to draw definitive conclusions for offline approaches. As illustrated by the temporal trend of RB through Table 1 online models—particularly those without RB—are reported more frequently. The increase observed around 2023 may reflect a growing focus on real-time applications in surgical environments. However, this observation may also be influenced by the uneven distribution of studies across years. Overall, offline models are less frequently reported, which may partially explain the limited use of RB in this setting.

From a methodological perspective, the use of RB introduces several implications. By allowing temporal tolerance at phase transitions, it may become less transparent at which points the model exhibits weaknesses. As a result, the diagnostic value of the evaluation may be reduced, particularly with respect to targeted model improvements. Furthermore, the inconsistent use of RB could complicate comparisons across studies, as models evaluated with and without such tolerance cannot be directly compared. This lack of consistency hinders the standardization of evaluation practices. Models evaluated with RB often may report higher performance values compared to those without RB. However, these results are frequently associated with higher standard deviations, indicating increased variability. This may suggest that RB acts as a methodological buffer, potentially inflating performance estimates while reducing the precision of evaluation. Consequently, the interpretability of model performance may be affected. RB may be particularly suitable in situations where phase transitions are inherently ambiguous, such as the transition from Calot's triangle dissection to clipping. However, in real-time safety applications, even short delays may be clinically relevant if alerts are intended to occur before irreversible actions, such as ductal clipping. In such scenarios, temporal tolerance may improve evaluation results while potentially masking clinically important timing errors. One possible approach could be to apply RB primarily in online models rather than in offline models, where real-time constraints may justify a certain degree of tolerance at phase transitions. This may help reduce false alarms and improve practical usability. At the same time, a more consistent and standardized use of evaluation metrics—particularly in online settings—could improve comparability across studies and support more reliable interpretation of results.

Figures 7, 8 indicate that, in both online and offline settings, the evaluation metrics accuracy, precision, recall, and jaccard index are most frequently used. Also the Table 1 further shows that, from 2023 onwards, segment-based metrics are increasingly reported. This may suggest a shift in the requirements of SPR models from purely classification-based objectives toward a stronger consideration of temporal dynamics. This development may also indicate an extension of existing evaluation practices. Also shown in both figures, segment-based metrics are more frequently used in online models, whereas their use in offline models is limited or absent. This observation may support the assumption that online phase recognition involves higher complexity and a stronger need to minimize false alarms. Despite the differing objectives of online and offline systems, the choice of evaluation metrics does not differ substantially between the two settings. This may be attributed to the lack of an established mapping between specific evaluation metrics and particular application scenarios. For example, precision may be more relevant for online models, where accurate predictions with a low false alarm rate are critical. In contrast, recall may play a more important role in offline scenarios, where the goal is to achieve a complete identification of all phases during retrospective analysis. From a clinical perspective, a high precision may be important to avoid false alarms that could distract the surgical team. In contrast, applications like documentation, workflow analysis may benefit more from high recall. Based on this, the *f*_1_-score could be adapted accordingly. For online scenarios, an *f*_β_-score with a stronger emphasis on precision may be more appropriate, whereas for offline scenarios, an *f*_β_-score that prioritizes recall could be more suitable. Segment-based metrics and jaccard index may be especially relevant for staff coordination and operating room management, where accurate timing is essential. Consequently, different application scenarios may require different evaluation priorities. So no single metric may be universally optimal for all clinical use cases.

Such a differentiated application of evaluation metrics could help align metric selection more closely with the requirements of specific application scenarios, whether online or offline. This would enable more methodologically consistent and meaningful comparisons within each category. In the long term, this approach could contribute to a more structured standardization of evaluation practices, allowing for both differentiated and comparable assessments in SPR. This observation is further supported by the fact that most metric combinations tend to include at least accuracy, precision, and recall. These metrics appear consistently across both online and offline models and over all years, suggesting a tendency toward standardization. An interesting observation is that the simultaneous use of the jaccard index and the *f*_1_-score is not reported in either online or offline settings. This may be explained by the differing objectives of these metrics. While the *f*_1_-score combines precision and recall—capturing both reliability and completeness—the jaccard index focuses on the overlap between predictions and GT, thereby emphasizing similarity between predicted and actual segments.

This difference in focus may lead to the selection of one metric over the other, depending on the evaluation objective. The jaccard index may be preferred in scenarios where a more direct comparison between prediction and GT is desired, which could explain its relatively frequent use in both online and offline settings. Based on this observation, an adaptation of the *f*-score, such as the *f*_β_-score, may provide a more flexible and task-specific evaluation by allowing different weightings of precision and recall.

In general, the fact that accuracy is the most commonly used evaluation metric in the SPR is at odds with theoretical considerations, as a key drawback of this metric is that it can obscure errors and be misleading in the presence of class imbalance. More specifically, accuracy may be misleading because long phases, such as gallbladder dissection, dominate the video material, whereas shorter phases, such as Calot's triangle dissection, are safety-critical. Consequently, due to this class imbalance, a model may achieve high overall accuracy despite performing poorly on clinically relevant shorter phases. Since SPR datasets consist exclusively of video data, the evaluation results may therefore be biased and fail to accurately reflect model performance. To more appropriately account for errors in imbalanced datasets, the *f*_1_-score would be preferable, as it combines the objectives of precision and recall. Particularly in the context of SPR, this metric could be advantageous, given the inherently imbalanced nature of video data. Nevertheless, the reviewed literature shows that this metric is not frequently used and therefore does not rank among the leading evaluation metrics in SPR. One possible explanation is that researchers tend to focus on reporting precision and recall separately, rather than using the *f*_1_-score as a combined measure.

No clear association between employed data splits and reported evaluation metrics could be identified in this field of SPR. Although several studies adopted similar metrics, no direct relationship between metric selection and model comparison was observed. Consequently, a straightforward comparison of models across studies is limited, as differences in both dataset partitioning and evaluation protocols hinder a direct assessment of performance. Therefore, results reported in the literature should be interpreted with caution when comparing models from different studies.

Furthermore, the MCC represents a potentially robust evaluation metric for SPR. Its extension to multi-class problems allows its application beyond binary classification tasks. In particular, MCC may be advantageous for imbalanced datasets and complex surgical procedures, such as laparoscopic cholecystectomy, which involve multiple phases with varying durations and levels of difficulty. Unlike individual metrics such as accuracy, precision, recall, the *f*_1_-score, or the jaccard index, MCC considers all entries of the confusion matrix and therefore provides a more balanced assessment of model performance. Consequently, MCC may complement rather than replace established evaluation metrics by providing additional information about the overall quality of classification. This may be particularly beneficial in SPR, where class imbalance is inherent due to varying phase durations. For example, long phases such as gallbladder dissection often dominate the video data, whereas shorter but safety-critical phases are comparatively underrepresented. Consequently, high accuracy values may still be achieved despite poor recognition of clinically important phases. By taking all classes into account simultaneously, MCC may provide a more comprehensive evaluation and help avoid biased performance estimates. However, its value range of [–1, 1] differs from that of commonly used evaluation metrics in SPR, which are typically bounded within [0, 1]. This difference may complicate direct comparisons with existing metrics. Therefore, a normalization of the MCC could be considered to improve comparability. Such an approach may enable the integration of MCC into a more standardized evaluation framework for multi-class problems in SPR. While the *f*_1_-score provides a balanced assessment of precision and recall, it does not account for TN. Consequently, correctly rejected non-target phases are not reflected in the evaluation. In multi-class SPR, where phases such as preparation, clipping, and gallbladder dissection must be distinguished simultaneously, this may limit the overall assessment of model performance. In contrast, MCC considers all entries of the confusion matrix and therefore provides a more holistic evaluation of classification performance. This may be particularly beneficial in SPR, where class imbalance is inherent due to varying phase durations. Therefore, MCC may complement established metrics such as accuracy, precision, recall, the *f*_1_-score, and the jaccard index by providing additional information about the overall quality of classification. Segment-based metrics may be particularly relevant for workflow coordination and operating room management, as they explicitly account for the temporal relationships between surgical phases. Similarly, the jaccard index focuses on the overlap between predictions and GT, thereby emphasizing segmentation quality rather than the overall distribution of classification errors. Consequently, neither segment-based metrics nor the jaccard index provide a comprehensive assessment of overall classification performance. In contrast, MCC considers all entries of the confusion matrix and therefore captures the overall quality of classification. Thus, MCC may represent a valuable complement to established evaluation metrics in SPR by providing additional information about the global distribution of prediction errors.

### 4.2 Limitations and future work

This paper is based on a literature review and is therefore subject to several limitations. First, the analysis was restricted to articles using the Cholec80 dataset, which limits the generalizability of the findings to other datasets and application domains. In addition, the number of articles identified within the selected search period was relatively small and unevenly distributed across the years, resulting in a heterogeneous body of literature. Consequently, the available evidence does not allow for statistical analyses that could further strengthen the observed trends and findings.

Another limitation concerns the imbalance between offline and online models, as only a limited number of articles investigated online approaches. This discrepancy prevents a direct and comprehensive comparison between both model settings. Therefore, future research should build upon these observations and perform statistical analyses to validate the reported findings and improve the transparency and robustness of the conclusions.

To improve the interpretability and comparability of results, future articles should explicitly explain and clearly document their choice of evaluation metrics. In addition, the use of RB should be consistently reported, as their inclusion can significantly influence performance outcomes and hinder direct comparisons with models that do not employ such tolerance. For a more standardized evaluation framework, future work should place greater emphasis on distinguishing between online and offline phase recognition settings. In this context, the selection of evaluation metrics could be aligned more closely with the respective application scenarios. For instance, precision may be prioritized in online settings, where minimizing false alarms is critical, whereas recall may be more relevant in offline scenarios, where completeness of phase detection is essential.

Accordingly, extensions of the *f*_1_-score, such as the *f*_β_-score, could be adapted to better reflect these differing priorities by weighting precision and recall according to the specific requirements of each setting. Such a differentiated approach may contribute to more consistent evaluation practices and facilitate more meaningful comparisons across articles. In general, it would be beneficial for future work to make more frequent use of the *f*_1_-score, as it may be more suitable for imbalanced datasets such as those in the SPR. The emphasis on accuracy should be reduced, as despite its simplicity, it can be misleading in the presence of class imbalance.

Furthermore, a normalization of the MCC metric is recommended to align it with standardized evaluation frameworks. Its integration into SPR could further enhance the comparability of models in multi-class settings.

As more articles become available, future work should include more comprehensive statistical analyses to investigate potential relationships between model architectures and the reported evaluation metrics. Such analyses could provide further insights into whether specific types of models are preferentially associated with particular evaluation protocols and improve the comparability of studies.

In summary, a well-structured approach should be followed in the SPR when selecting and explaining evaluation metrics. The primary objective according to which the model is to be evaluated should be clearly defined and consistently taken into account.

## References

[B1] Ban Y. Rosman G. Eckhoff J. A. Ward T. M. Hashimoto D. A. Kondo T. . (2022). SUPR-GAN: surgical prediction gan for event anticipation in laparoscopic and robotic surgery. IEEE Robot. Autom. Lett. 7, 5741–5748. doi: 10.1109/LRA.2022.3156856

[B2] Ban Y. Rosman G. Ward T. Hashimoto D. Kondo T. Iwaki H. . (2021). “Aggregating long-term context for learning laparoscopic and robot-assisted surgical workflows,” in 2021 IEEE international conference on robotics and automation (ICRA) (Xi'an), 14531–14538. doi: 10.1109/ICRA48506.2021.9561770

[B3] Chen H.-B. Li Z. Fu P. Ni Z.-L. Bian G.-B. (2022). “Spatio-temporal causal transformer for multi-grained surgical phase recognition,” in 2022 44th Annual international conference of the IEEE engineering in medicine and biology society (EMBC) (Glasgow), 1663–1666. doi: 10.1109/EMBC48229.2022.9871004 36086459

[B4] Cheng K. You J. Wu S. Chen Z. Zhou Z. Guan J. . (2022). Artificial intelligence-based automated laparoscopic cholecystectomy surgical phase recognition and analysis. Surg. Endosc. 36, 3160–3168. doi: 10.1007/s00464-021-08619-3 34231066

[B5] Czempiel T. Paschali M. Ostler D. Kim S. T. Busam B. Navab N. (2020). “TeCNO: surgical phase recognition with multi-stage temporal convolutional networks,” in Medical Image Computing and Computer Assisted Intervention-MICCAI 2020, Volume 12263 of Lecture Notes in Computer Science, eds. A. L. Martel, P. Abolmaesumi, D. Stoyanov, D. Mateus, M. A. Zuluaga, S. K. Zhou, et al. (Cham: Springer), 343–352. doi: 10.1007/978-3-030-59716-0_33

[B6] Czempiel T. Paschali M. Ostler D. Kim S. T. Busam B. Navab N. (2021). “OperA: attention-regularized transformers for surgical phase recognition,” in Medical Image Computing and Computer Assisted Intervention-MICCAI 2021, Volume 12904 of Lecture Notes in Computer Science, eds. M. de Bruijne, P. C. Cattin, S. Cotin, N. Padoy, S. Speidel, Y. Zheng, et al. (Cham: Springer), 604–614. doi: 10.1007/978-3-030-87202-1_58

[B7] De Backer P. Peraire Lores M. Demuynck M. Piramide F. Simoens J. Oosterlinck T. . (2023). Surgical phase duration in robot-assisted partial nephrectomy: a surgical data science exploration for clinical relevance. Diagnostics 13:3386. doi: 10.3390/diagnostics13213386 37958283 PMC10650909

[B8] Demir K. C. Lojo Rodríguez B. Weise T. Maier A. Yang S. H. (2024). “Towards intelligent speech assistants in operating rooms: a multimodal model for surgical workflow analysis,” in Proceedings of Interspeech 2024 (Kos), 1465–1469. doi: 10.21437/Interspeech.2024-975

[B9] Demir K. C. Schieber H. Weise T. Roth D. May M. Maier A. . (2023). Deep learning in surgical workflow analysis: a review of phase and step recognition. IEEE J. Biomed. Health Inf. 27, 5405–5417. doi: 10.1109/JBHI.2023.3311628 37665700

[B10] Ding X. Li X. (2022). Exploring segment-level semantics for online phase recognition from surgical videos. IEEE Trans. Med. Imaging 41, 3309–3319. doi: 10.1109/TMI.2022.3182995 35700259

[B11] Funke I. Rivoir D. Krell S. Speidel S. (2025). TUNeS: a temporal U-Net with self-attention for video-based surgical phase recognition. IEEE Trans. Biomed. Eng. 72, 2105–2119. doi: 10.1109/TBME.2025.3535228 40031317

[B12] Funke I. Rivoir D. Speidel S. (2023). Metrics matter in surgical phase recognition. arXiv [Preprint], arXiv:2305.13961.

[B13] Gao X. Jin Y. Long Y. Dou Q. Heng P.-A. (2021). “Trans-SVNet: accurate phase recognition from surgical videos via hybrid embedding aggregation transformer,” in Medical Image Computing and Computer Assisted Intervention-MICCAI 2021, Volume 12904 of Lecture Notes in Computer Science, eds. M. de Bruijne, P. C. Cattin, S. Cotin, N. Padoy, C. Speidel, et al. (Berlin: Springer), 593–603. doi: 10.1007/978-3-030-87202-1_57

[B14] Hicks S. A. Strümke I. Thambawita V. Hammou M. Riegler M. A. Halvorsen P. . (2022). On evaluation metrics for medical applications of artificial intelligence. Sci. Rep. 12:5979. doi: 10.1038/s41598-022-09954-8 35395867 PMC8993826

[B15] Jin Y. (2018). SV-RCNet Surgical Video Evaluation (Cholec80). Available online at: https://github.com/YuemingJin/SV-RCNet/tree/8df7e10f7cf26b8ef1eb05b2c73d1f4020393a49/surgicalVideo/evaluation_Cholec80 (Accessed July 13, 2025).

[B16] Jin Y. Li H. Dou Q. Chen H. Qin J. Fu C.-W. . (2020). Multi-task recurrent convolutional network with correlation loss for surgical video analysis. Med. Image Anal. 59:101572. doi: 10.1016/j.media.2019.101572 31639622

[B17] Jin Y. Long Y. Chen C. Zhao Z. Dou Q. Heng P.-A. (2021). Temporal memory relation network for workflow recognition from surgical video. IEEE Trans. Med. Imaging 40, 1911–1923. doi: 10.1109/TMI.2021.3069471 33780335

[B18] Kadkhodamohammadi A. Luengo I. Stoyanov D. (2022). PATG: position-aware temporal graph networks for surgical phase recognition on laparoscopic videos. Int. J. Comput. Assist. Radiol. Surg. 17, 849–856. doi: 10.1007/s11548-022-02600-8 35353299

[B19] Lee S.-G. Kim G.-Y. Hwang Y.-N. Kwon J.-Y. Kim S.-M. (2024). Adaptive undersampling and short clip-based two-stream CNN-LSTM model for surgical phase recognition on cholecystectomy videos. Biomed. Signal Process. Control 88:105637. doi: 10.1016/j.bspc.2023.105637

[B20] Li Y. Li Y. He W. Shi W. Wang T. Li Y. (2021). “SE-OHFM: a surgical phase recognition network with SE attention module,” in 2021 international conference on electronic information engineering and computer science (EIECS) (Changchun), 608–611. doi: 10.1109/EIECS53707.2021.9587961

[B21] Li Y. Zhao Z. Li R. Li F. (2024). Deep learning for surgical workflow analysis: a survey of progresses, limitations, and trends. Artif. Intell. Rev. 57:291. doi: 10.5772/intechopen.111293

[B22] Liu Y. Boels M. Garcia-Peraza-Herrera L. C. Vercauteren T. Dasgupta P. Granados A. . (2025). Lovit: long video transformer for surgical phase recognition. Med. Image Anal. 99:103366. doi: 10.1016/j.media.2024.103366 39418831 PMC11876726

[B23] Liu Y. Huo J. Peng J. Sparks R. Dasgupta P. Granados A. . (2023). “SKiT: a fast key information video transformer for online surgical phase recognition,” in Proceedings of the IEEE/CVF international conference on computer vision (ICCV) (Paris), 21017–21027. doi: 10.1109/ICCV51070.2023.01927

[B24] Mishra V. Mishra M. P. (2023). “Prisma for review of management literature: method, merits, and limitations: an academic review,” in Advancing Methodologies of Conducting Literature Review in Management Domain, eds. S. Rana, J. Singh, and S. Kathuria (Bingley: Emerald Publishing Limited), 125–136. doi: 10.1108/S2754-586520230000002007

[B25] Naidu G. Zuva T. Sibanda E. M. (2023). “A review of evaluation metrics in machine learning algorithms,” in Artificial Intelligence Application in Networks and Systems, Volume 724 of Lecture Notes in Networks and Systems, eds. R. Silhavy, and P. Silhavy (Cham: Springer International Publishing), 15–25. doi: 10.1007/978-3-031-35314-7_2

[B26] Pan X. Gao X. Wang H. Zhang W. Mu Y. He X. (2023). Temporal-based swin transformer network for workflow recognition of surgical video. Int. J. Comput. Assist. Radiol. Surg. 18, 139–147. doi: 10.1007/s11548-022-02785-y 36331795

[B27] Pradeep C. S. Sinha N. (2021). “Spatio-temporal features based surgical phase classification using CNNs,” in 2021 43rd annual international conference of the IEEE engineering in medicine biology society (EMBC) (Mexico), 3332–3335. doi: 10.1109/EMBC46164.2021.9630829 34891953

[B28] Putalapattu P. (2022). “DORC: surgical workflow recognition tool using fast R-CNN and modified HMMS,” in Proceedings of the future technologies conference (FTC) 2021, ed. K. Arai (Cham: Springer International Publishing), 636–644. doi: 10.1007/978-3-030-89880-9_47

[B29] Sasaki Y. (2007). “The truth of the f-measure,” in Proceedings of the workshop on machine learning and data mining in pattern recognition (MLDM) (Leipzig).

[B30] Tao R. Zou X. Zheng G. (2023). LAST: latent space-constrained transformers for automatic surgical phase recognition and tool presence detection. IEEE Trans. Med. Imaging 42, 3256–3268. doi: 10.1109/TMI.2023.3279838 37227905

[B31] Terven J. Cordova-Esparza D.-M. Romero-González J.-A. Ramírez-Pedraza A. Chávez-Urbiola E. A. (2025). A comprehensive survey of loss functions and metrics in deep learning. Artif. Intell. Rev. 58:195. doi: 10.1007/s10462-025-11198-7

[B32] Twinanda A. P. Shehata S. Mutter D. Marescaux J. De Mathelin M. Padoy N. (2017). EndoNet: a deep architecture for recognition tasks on laparoscopic videos. IEEE Trans. Med. Imaging 36, 86–97. doi: 10.1109/TMI.2016.2593957 27455522

[B33] Yi F. Jiang T. (2019). “Hard frame detection and online mapping for surgical phase recognition,” in Medical Image Computing and Computer Assisted Intervention-MICCAI 2019, Volume 11768 of Lecture Notes in Computer Science, eds. D. Shen, T. Liu, T. M. Peters, L. H. Staib, C. Essert, S. Zhou, P.-T. Yap, and A. Khan (Cham: Springer), 449–457. doi: 10.1007/978-3-030-32254-0_50

[B34] Yi F. Yang Y. Jiang T. (2023). “Not end-to-end: explore multi-stage architecture for online surgical phase recognition,” in Computer Vision-ACCV 2022, volume 13844 of Lecture Notes in Computer Science, eds. L. Wang, J. Gall, T.-J. Chin, I. Sato, and R. Chellappa (Cham: Springer), 417–432. doi: 10.1007/978-3-031-26316-3_25

[B35] Yue W. Liao H. Xia Y. Lam V. Luo J. Wang Z. (2023). Cascade multi-level transformer network for surgical workflow analysis. IEEE Trans. Med. Imaging 42, 2817–2831. doi: 10.1109/TMI.2023.3265354 37037257

[B36] Zhang B. Fung A. Torabi M. Barker J. Foley G. Abukhalil R. . (2023). “C-ECT: online surgical phase recognition with cross-enhancement causal transformer,” in 2023 IEEE 20th International Symposium on Biomedical Imaging (ISBI) (Cartagena de Indias), 1–5. doi: 10.1109/ISBI53787.2023.10230841

[B37] Zhang B. Ghanem A. Simes A. Choi H. Yoo A. (2021). Surgical workflow recognition with 3D CNN for sleeve gastrectomy. Int. J. Comput. Assist. Radiol. Surg. 16, 2029–2036. doi: 10.1007/s11548-021-02473-3 34415503 PMC8589754

[B38] Zhang B. Meng J. Cheng B. Biskup D. Petculescu S. Chapman A. (2024a). “Friends across time: multi-scale action segmentation transformer for surgical phase recognition,” in 2024 annual international conference of the IEEE engineering in medicine and biology society (EMBC) (Orlando, FL), 1–6. doi: 10.1109/EMBC53108.2024.10782887 40039574

[B39] Zhang B. Sarhan M. H. Goel B. Petculescu S. Ghanem A. (2024b). SF-TMN: Slowfast temporal modeling network for surgical phase recognition. Int. J. Comput. Assist. Radiol. Surg. 19, 871–880. doi: 10.1007/s11548-024-03095-1 38512588

[B40] Zhang S. Xu T. Cao Z. Liao H. Ning G. Chen F. (2024c). “Frequency-based temporal analysis network for accurate phase recognition from surgical videos,” in 2024 IEEE international symposium on biomedical imaging (ISBI) (Athens), 1–5. doi: 10.1109/ISBI56570.2024.10635806

[B41] Zou X. Liu W. Wang J. Tao R. Zheng G. (2023). ARST: auto-regressive surgical transformer for phase recognition from laparoscopic videos. Comput. Methods Biomech. Biomed. Eng. Imaging Vis. 11, 1012–1018. doi: 10.1080/21681163.2022.2145238

